# Metagenome-based virome analysis identifies the oral viral signatures for periodontitis

**DOI:** 10.1080/20002297.2026.2662091

**Published:** 2026-04-25

**Authors:** Tianshu Chu, Jian Liu, Yue Zhang, Kai Yang, Shenghui Li, Qiulong Yan, Yuhong Li

**Affiliations:** a State Key Laboratory of Oral & Maxillofacial Reconstruction and Regeneration, Key Laboratory of Oral Biomedicine Ministry of Education, Hubei Key Laboratory of Stomatology, School & Hospital of Stomatology, Wuhan University, Wuhan, China; b Puensum Genetech Institute, Wuhan, China; c College of Veterinary Medicine, Qingdao Agricultural University, Qingdao, China; d Department of Microbiology, Department of Biochemistry and Molecular Biology, College of Basic Medical Sciences, Dalian Medical University, Dalian, China; e The Fifth Affiliated Hospital of Southern Medical University, Guangzhou, China

**Keywords:** Periodontitis, oral virome, bacteriophage, metagenomics, biomarker, function annotation

## Abstract

**Background:**

Periodontitis (PD) is a chronic infectious disease driven by bacterial biofilms, yet the oral virome's role in pathogenesis remains poorly understood.

**Objective:**

This cross-cohort meta-analysis aims to define PD-associated viral signatures, characterize predicted virus-host interactions, and evaluate the diagnostic potential of viral biomarkers.

**Methods:**

We integrated 89 saliva (44 PD, 45 healthy) and 86 subgingival plaque (48 PD, 38 healthy) metagenomes from six public cohorts for a unified virome analysis.

**Results:**

We identified 156 viral operational taxonomic units (vOTUs) significantly associated with PD (105 in saliva, 66 in subgingival plaque and 15 shared). PD-enriched vOTUs were predicted to target periodontal pathogens including *Porphyromonas gingivalis*, whereas *Streptococcus*-targeting phages were decreased. PD-associated vOTUs harbored diverse bacterial defense and anti-defense systems, with those enriched in PD overrepresenting lysozyme and replication-associated genes. Diagnostic models based on key viral markers achieved robust performance, with AUCs of 0.95 (saliva) and 0.92 (subgingival plaque) for classifying PD.

**Conclusion:**

This study delineates a distinct oral virome profile in PD, highlights predicted virus-host interactions, and underscores the potential of viral biomarkers for PD diagnosis,providing a basis for future investigations into viral ecology and phage-based interventions.

## Key messages

- The oral virome undergoes a significant and consistent shift in periodontitis, characterized by the increase of bacteriophages targeting periodontal pathogens and the reduction of others. The PD-associated oral vOTUs are predominantly temperate and harbor a diverse arsenal of defense and anti-defense systems, with the subset enriched in disease exhibiting significant overrepresentation of lysozyme and a parallel trend for DNA replication-associated genes, suggesting a complex interplay with bacterial hosts in the periodontal niche. Viral biomarkers demonstrate promising preliminary potential as non-invasive diagnostic aids for periodontitis.

## Graphical abstract

**Figure:**
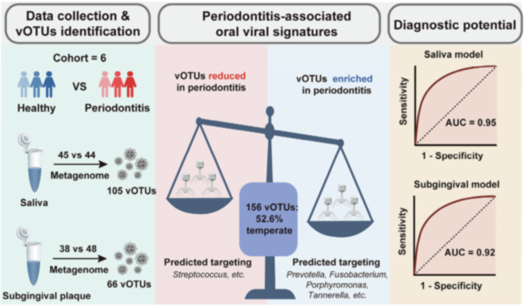

## Introduction

Periodontitis (PD) is a common disease affecting the human oral cavity, impacting the supporting structures of teeth, including the gingiva, periodontal ligament and alveolar bone [1]. According to a systematic review, between 2011 and 2020, the estimated pooled prevalence of PD among dentate adults (18 years old or older) across 17 countries was approximately 62%, with its severe stage affecting approximately 23.6% of the studied population, indicating a remarkably high prevalence compared to previous estimates from 1990 to 2010 [2]. PD significantly impacts oral health and serves as the leading cause of tooth loss in the elderly [3]. Moreover, it is closely linked to systemic diseases such as cardiovascular disease and diabetes, underscoring the importance of periodontal health for overall well-being [4].

Currently, PD is recognised as a multifactorial condition, with its pathogenesis involving a dynamic interplay between bacterial biofilms and the host immune response [5]. Additionally, the composition of the microbial community in the subgingival area is indicative of the severity and progression of periodontal disease, where dysbiosis—an imbalance in microbial flora—significantly contributes to disease onset and advancement [6]. Specific bacterial species within the dental plaque biofilm, such as *Porphyromonas gingivalis*, *Tannerella forsythia* and *Treponema denticola*, are recognised as key pathogens in the development of PD and collectively form the ‘red complex’, a proposed pathogenic consortium responsible for PD progression [7].

Viruses, in addition to bacteria, are crucial members of the human oral microbial ecosystem, and the oral virome represents an essential, yet long-overlooked, component of the human oral microbiome [8]. Various eukaryotic viruses, including human cytomegalovirus (HCMV), Epstein-Barr virus (EBV) and herpes simplex virus (HSV), have been detected in the periodontal tissues and saliva of patients with PD, showing associations with the disease [9,10]. Beyond these eukaryotic viruses, bacteriophages have also emerged as important players in oral ecology. Evidence indicates that prophages are common in *Porphyromonas gingivalis*, exhibiting considerable genomic diversity and encoding genes with the potential to alter the physiology and interactions of this keystone pathogen [11]. Critically, despite their therapeutic potential for dysbiosis-driven pathologies, phage-based interventions have not been rigorously evaluated in PD [12]. Thus far, only a limited number of studies have been implicated in the oral virome associated with PD. Early metagenomic sequencing by Wang et al. identified *Actinomyces* phage as the most abundant type in plaque samples from periodontally healthy individuals [13]. Complementing this, a study employed metagenomic sequencing on passive drool samples from healthy individuals in North Carolina, USA, to characterise the human oral DNA virome, identifying diverse *Anelloviruses*, *Cressdnaviruses* and bacteriophages [14]. Recently, Ye et al. reported significant alterations in oral viral communities in PD patients and/or hypertension, identifying disease-associated viral signatures and revealing potential virus‒bacterium interactions that may contribute to periodontal dysbiosis and systemic hypertension [15]. Significantly, Xu et al. combined *in silico* screening of six putative bacteriophage-derived endolysins targeting *Porphyromonas gingivalis* with experimental validation, demonstrating that a mixture of three endolysins significantly inhibits *Porphyromonas gingivalis* growth *in vitro* and highlights their therapeutic potential for PD with type 2 diabetes mellitus [16]. Given the current lack of large-scale and cross-cohort studies on the oral virome related to PD, conducting meta-analyses is essential to avoid biased associations between the oral virome and PD.

In this study, we integrated 234 oral metagenomes from six published cohorts to profile the oral virome and identify viral signatures associated with PD. Adopting a methodology previously applied to gut virome studies in colorectal cancer [17], we performed meta-analyses to identify reliable PD-associated oral viral signatures. Our analysis focused on characterising the genomic features of these viruses and their predicted interaction with potential bacterial hosts. Additionally, to evaluate the diagnostic potential of these viral signatures, we constructed and validated random forest models using intra-dataset, cross-dataset and leave-one-cohort-out (LOCO) strategies.

## Methods

### Ethics statement

The metagenomic data were obtained from the National Centre for Biotechnology Information (NCBI) Sequence Read Archive (SRA), originating from prior studies that received ethical approval and informed consent in accordance with the Declaration of Helsinki. As all data were de-identified, no additional ethics approval was required for this secondary analysis.

### Data collection

Publicly available datasets from six PD studies were downloaded from the NCBI SRA, encompassing oral metagenomes from 117 patients with PD and 107 healthy controls. The following accession IDs were: PRJNA230363 for Wang_2015 [13], PRJNA396840 for Belstrøm_2017 [18], PRJDB11203 for Izawa_2021 [19], PRJNA678453 for Belstrøm_2021 [20,21], PRJNA717815 for Saito_2022 [22,23] and PRJNA932552 for Moghadam_2023 [24]. Clinical diagnostic criteria and sample details were summarised in Table S1. Although specific criteria were not explicitly stated for Saito_2022, it was included based on its explicit case‒control design. Samples from individuals with caries (*n* = 10) were excluded from all subsequent comparative analyses between PD and healthy controls to avoid potential confounding, and different oral sites were analysed separately.

### Data preprocessing

Raw paired-end metagenomic reads underwent quality control using fastp v0.20.1 with the options ‘*-u 30 -q 20 -l 60 -y -Y 30 –trim_poly_g*’ [25]. To remove host-derived sequences, quality-filtered reads were aligned to the human reference genome (CHM13v2.0) using Bowtie2 v2.4.1 with the parameters ‘*--end-to-end --fast --no-head*’ [26]. The unmapped read pairs were retained as clean reads for subsequent analyses.

### Taxonomic profiling

Clean reads were aligned to the reference sequences of viral operational taxonomic units (vOTUs) from the Oral Virus Database (OVD) using Bowtie2 v2.4.1 with the parameters ‘*--end-to-end --fast --no-unal’* [27]. Raw read counts per vOTU were normalised to transcripts per million (TPM) to account for vOTU length and sequencing depth [28]. To minimise noise from spurious or low-abundance detections, a prevalence filter was applied: a vOTU was considered reliably present in a cohort only if it achieved a TPM value of at least 0.1 in no fewer than 10% of the samples within that cohort. For each sample, the abundance of vOTUs failing to meet this threshold was set to zero. The 10% prevalence threshold was applied as a relative criterion to ensure comparable filtering stringency across cohorts of unequal sample sizes, thereby avoiding the disproportionate exclusion of vOTUs from smaller cohorts that would result from a fixed absolute count threshold. Alternative thresholds were considered, but either introduced greater sensitivity imbalance or failed to adequately suppress noise in smaller datasets. Viral family-level abundances were calculated by summing the TPM values of all vOTUs classified under the same family. Sequentially, bacterial community composition was analysed from the same set of clean reads using MetaPhlAn v4.0 with its default database and parameters [29].

### Diversity of oral viral community in relation to PD

Alpha diversity was assessed at the vOTU level based on relative abundance profiles using two metrics: the Shannon index and the observed number of vOTUs. The Shannon index was calculated using the *diversity* function with the option *index = shannon* in R. We separately annotated the bacterial communities using the same metrics, with bacterial diversity assessed at the species level.

Beta diversity was evaluated on Hellinger-transformed abundances using Bray–Curtis distances. Principal coordinate analysis (PCoA) was performed with the *cmdscale* function (*ape* package) to visualise community structure. PERMANOVA was conducted using the *adonis2* function (*vegan* package) with 999 permutations, stratifying by cohort (*strata = project*).

### Identification of PD-associated viral biomarkers in saliva and subgingival plaque

To identify robust viral biomarkers, our analysis focused on saliva and subgingival plaque due to their established relevance to PD pathogenesis and the availability of sufficient cross-cohort data. Saliva provides an integrated profile of oral microbial and inflammatory activity, whereas subgingival plaque harbours the dysbiotic biofilm directly associated with tissue destruction [30,31]. Samples were excluded based on: (1) limited cohort availability (tongue and supragingival plaque, available in only one or two cohorts); (2) absence of case‒control pairing (saliva from Wang_2015, containing only controls).

Viral biomarker discovery was therefore conducted using the four remaining saliva datasets and four subgingival plaque datasets. Within each cohort, vOTU abundances were compared between PD patients and healthy controls using the Wilcoxon rank-sum test. We retained vOTUs that were detected in at least two cohorts and showed a consistent direction with *p* < 0.05, resulting in 202 candidate vOTUs from saliva and 123 from subgingival plaque for subsequent meta-analysis.

### Meta-analysis

To synthesise evidence across the independent cohorts and formally identify robust PD-associated signatures, we applied a random-effects meta-analysis to the candidate vOTUs. For each candidate vOTU, TPM values were log10-transformed after adding a small constant (minimum positive abundance). Random-effects meta-analysis was performed using the *escalc* (*measure = SMD*) and *rma* functions in R to compute Hedges' g effect sizes with 95% confidence intervals. Inter-study heterogeneity was assessed via Cochran's Q test and I² statistics. *P-values* were adjusted by the Benjamini–Hochberg procedure. vOTUs with meta-analysis BH-adjusted *P*-value < 0.05 were considered significantly associated with PD, yielding a final set of 105 salivary and 66 subgingival plaque biomarkers.

### Confounding assessment

For cohorts with individual-level demographic data (saliva: Belstrøm_2021, Moghadam_2023; subgingival plaque: Belstrøm_2021, Moghadam_2023, Izawa_2021), we compared unadjusted Wilcoxon *P*-values with those from linear models adjusting for age, sex, or both. A vOTU was considered robust if it remained significant (*P* < 0.05) with consistent direction after adjustment.

### Permutation test

To evaluate false positive risk, disease labels were randomly shuffled within each cohort (preserving sample sizes), and the entire biomarker discovery pipeline (within-cohort Wilcoxon, consistency filter, meta-analysis) was reapplied. This process was repeated 1,000 times to generate null distributions; empirical *P*-values were calculated as (number of permutations with ≥observed biomarkers + 1)/(1,000 + 1).

### Virus-host prediction

Virus-host interactions were predicted using two complementary sequence-based methods: CRISPR-spacer matches and prophage blasts [27]. This analysis was based on the oral microbial genome catalogue, derived from 3,569 prokaryotic species (containing over 50,000 metagenome-assembled genomes) from a previous study [32]. For CRISPR-spacer matches, we firstly predicted CRISPR spacer sequences from the oral prokaryotic genome catalogue using MinCED v0.4.2 [33] with the option ‘*-minNR 2*’, and then assigned a host to the virus if host CRISPR spacer sequence was matched to the viral genome (bit-score ≥ 45) using BLASTn with options ‘*-evalue 1e-5 -word_size 8 -num_alignments 99999*’ [34]. For prophage blasts, the viral sequence was blasted against host genome sequences, and assigned a host if the viral sequence was exactly matched to the host genome at ≥90% nucleotide identity and ≥30% viral coverage [34].

Given the well-recognised challenges of correlation-based analyses in compositional microbiome data [35,36], which can lead to spurious associations, we employed these sequence-based methods to predict virus-host interactions rather than relying on abundance-based correlations. Based on these host assignments, we visualised the predicted virus-host association networks using Cytoscape v3.10.0 [37].

### Viral genomic analysis

vOTU quality and completeness were assessed with CheckV [38]. Viral open reading frames (ORFs) were predicted with Prodigal v2.6.3 in metagenomic mode (*-p meta*). Functional annotation was performed via DIAMOND v2.0.13 BLASTP searches against the KEGG Orthology (KO) database. To ensure robust homology-based annotation, hits to the KO database were retained only if they met thresholds of query coverage ≥50% and bit-score ≥60 [39]. BACPHLIP was employed for lifestyle prediction, classifying vOTUs as virulent or temperate based on a probability threshold of >0.5 [40]. DefenseFinder v2.0.0 (default parameters) was used to systematically identify genes associated with prokaryotic defence systems and anti-prokaryotic defence systems [41]. Phylogenetic reconstruction was performed using ViPTreeGen [42]. Resultant trees were visualised in tvBOT (https://www.chiplot.online/) [43]. Intergenomic similarity analysis was generated by VIRIDIC (https://rhea.icbm.uni-oldenburg.de/viridic/) [44].

### Random forest prediction modelling

Random forest classifiers were built using the *caret* package in R on raw vOTU abundances without preprocessing. The number of trees (*ntree*) was fixed at 2,000 to ensure model stability and convergence [17]. The hyperparameter *mtry* was tuned via cross‑validation using an adaptive grid: for ≤15 features, all values from 1 to the total number were evaluated; for >15 features, five equally spaced values from 1 to one-third of the total were tested. The *nodesize* parameter was left at its default value.

To address class imbalance, sampling strategies varied by dataset and validation scheme. For saliva, SMOTE was consistently applied within repeated 5-fold cross-validation (5 repeats). For subgingival plaque, an adaptive approach was used: when training sets had <20 total samples or ≤5 minority class samples, up-sampling combined with leave-one-out cross-validation (LOOCV) was applied; otherwise, SMOTE with repeated 5-fold cross-validation was used. A sensitivity analysis using uniform up‑sampling with LOOCV across all subgingival plaque cohorts confirmed that the adaptive sampling scheme had a negligible impact on model performance.

Model performance was primarily assessed by the area under the ROC curve (AUC) using *pROC*, with feature importance quantified by mean decrease in accuracy. For LOCO validations, we additionally report accuracy, sensitivity, specificity, PPV, NPV and F1-score, with confusion matrices provided in the supplementary materials.

In summary, three validation schemes were implemented: (i) intra-cohort: 5-times repeated 5-fold cross-validation within each dataset; (ii) cross-cohort: models trained on one cohort and tested on the remaining three; (iii) leave-one-cohort-out (LOCO): each cohort served once as the test set while the other three were combined for training.

### Statistical analysis and visualisation

Statistical analysis and visualisation were conducted in R v4.4.1, utilising the *ggplot2* (3.5.1) and *pheatmap* (1.0.12) packages.

## Results

### Dataset characteristics and viral profiling

We included six publicly available datasets from four different countries that utilised whole-metagenome shotgun sequencing, encompassing 234 oral samples (117 PD, 107 healthy controls and 10 caries) and totalling nearly 940 Gbp of sequence data (Table 1). The isolation sources included saliva, supragingival plaque, subgingival plaque and tongue. Additional information including sequencing platform and read length for each dataset was summarised in Table 1.

**Table 1.** Summary of sample characteristics from the datasets included in this study^**1**^

			Number of samples							
Dataset	Country	Isolation source	Caries	Healthy	Periodontitis	Data per sample (Gbp)	Total data amount(Gbp)	NCBI accession ID	Sequencing platform	Read Length
Wang_2015 [13]	China	Saliva	–	8	–	10.75	85.98	PRJNA230363	Illumina HiSeq 2000	2 × 100 bp
		Supragingival plaque	–	4	5	0.96	8.63			
		Subgingival plaque	–	6	5	1.12	12.40			
Belstrøm_2017 [18]	Denmark	Saliva	10	10	10	1.60	48.07	PRJNA396840	Illumina HiSeq 2500	DNA: 2 × 250 bp RNA: 2 × 100 bp
Izawa_2021 [19]	Japan	Subgingival plaque	–	19	23	0.58	24.47	PRJDB11203	Illumina MiSeq	2 × 150 bp
Belstrøm_2021 [20,21]	Denmark	Saliva	–	11	11	3.62	79.72	PRJNA678453	Illumina HiSeq 2500	DNA: 2 × 250 bp; RNA: 2 × 100 bp
		Subgingival plaque	–	10	9	3.90	74.18			
		Tongue	–	8	10	8.36	150.43			
Saito_2022 [22,23]	Brazil	Saliva	–	13	14	1.09	29.59	PRJNA717815	Illumina HiSeq 4000	2 × 150 bp
Moghadam_2023 [24]	China	Saliva	–	11	9	5.55	111.03	PRJNA932553	Illumina NovaSeq 6000	2 × 151 bp
		Supragingival plaque	–	4	10	13.60	190.38			
		Subgingival plaque	–	3	11	8.93	125.017			
Overall	–	–	10	107	117	4.02	939.92	–	–	–

^1^ The 10 caries patients were excluded from all PD vs. healthy control comparisons.

To characterise the oral viral communities, we generated a virome profile encompassing 37,692 viral vOTUs (see Methods). Taxonomically, 58.9% of all 37,692 vOTUs were assigned to viral families under the traditional morphological system, predominantly comprising the former families *Siphoviridae* (43.6%), *Myoviridae* (10.5%) and *Podoviridae* (3.2%). Note that in the current ICTV phylogenomic framework, these traditional morphological groups have been replaced by more than 40 phylogenomically defined families within the class *Caudoviricetes*, and the original family names are no longer valid taxonomic ranks.

**Figure 1.**
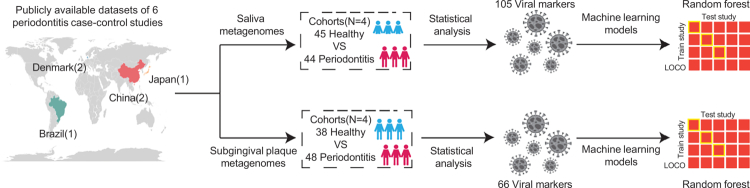
Study flowchart. Schematic overview of the study design, illustrating the global distribution of six publicly available datasets comprising both PD and healthy control cohorts, and the analytical workflow. Analyses were performed separately for saliva and subgingival plaque, including viral biomarker identification and random forest modelling.

Considering the influence of different oral cavity sites and the limited number of projects in supragingival plaque and tongue, we focused our subsequent analysis on saliva, which included 44 PD patients and 45 healthy controls (excluding 8 samples from healthy individuals in Wang_2015, which lacked a PD group), and subgingival plaque, comprising 48 PD patients and 38 healthy controls (Figure 1).

### Viral community diversity in PD

Alpha diversity metrics revealed site-specific alterations in PD. Observed number of vOTUs showed a consistent decreasing trend across salivary datasets, reaching significance in Saito_2022 (Wilcoxon, *P *= 0.001) and in the pooled salivary analysis (*P* = 0.004) (Figure S1A-B). Conversely, the Shannon index was significantly increased in pooled saliva samples (*P* = 0.001), driven mainly by Moghadam_2023 (*P* = 0.010) (Figure S1C-D). The discordant pattern observed in saliva, characterised by reduced vOTU richness alongside increased Shannon diversity, suggests that PD may drive the selective depletion of rare or low-abundance viral taxa while promoting greater evenness among the remaining dominant vOTUs. In subgingival plaque, the observed number of vOTUs exhibited a consistent downward trend across all four datasets (Figure S1A), while Shannon index changes were inconsistent, with a significant decrease only in Moghadam_2023 (*P* = 0.039) (Figure S1C). Notably, both alpha diversity metrics in saliva and subgingival plaque were highly consistent with their bacterial diversity, suggesting potential associations between the viral and bacterial communities (Figure S1E-F). Of note, our re-analysis of the bacterial communities using the MetaPhlAn4 database recapitulated the primary findings reported in the original individual studies (e.g. enrichment of classic periodontal pathogens in PD), with no substantial discrepancies observed. This concordance provides confidence in the overall data integrity and analytical approach.

**Figure 2.**
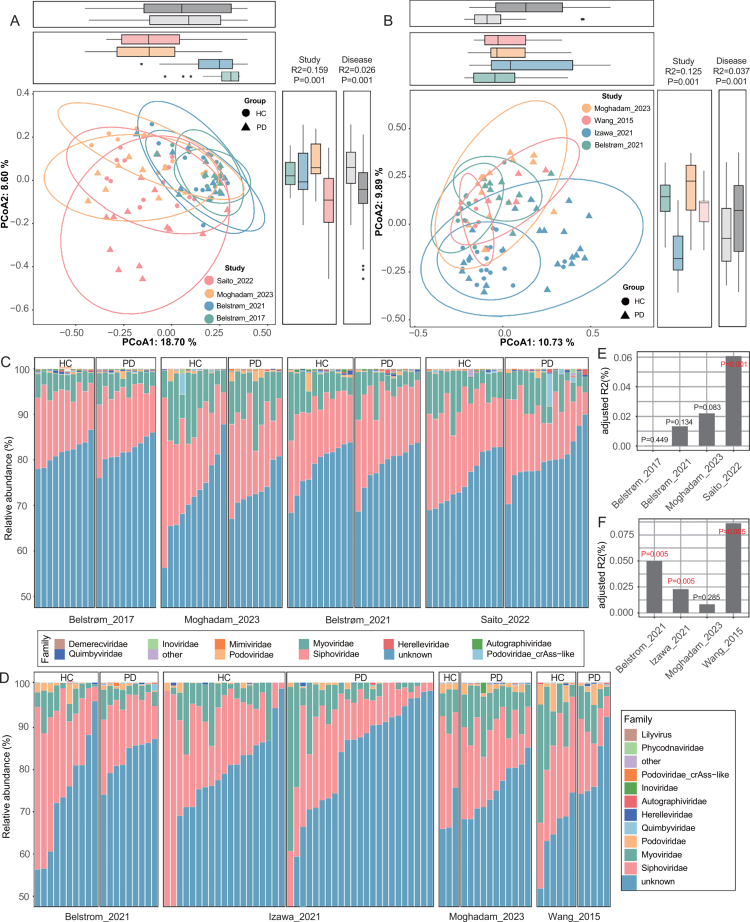
Viral community variation across cohorts and oral sites. (A–B) Principal coordinates analysis (PCoA) based on the Bray‒Curtis distance at the vOTU level in saliva (A) and subgingival plaque (B). Top and right margin boxplots show the distribution of PCoA1 and PCoA2 scores, respectively, stratified by study cohort (coloured boxes) and disease status (grey boxes). HC, healthy controls; PD, periodontitis patients. (C–D) The family-level composition of oral virome in each dataset in saliva (C) and subgingival plaque (D). (E–F) Effect size (adjusted R^2^) of PD status on viral community composition in each dataset for saliva (E) and subgingival plaque (F).

Beta diversity analysis using Bray-Curtis distances revealed significant separation by disease status in both sites after stratifying by cohort (PERMANOVA: saliva, *R²* = 2.6%, *P* = 0.001; subgingival plaque, *R²* = 3.7%, *P* = 0.001) (Figure 2A–B). The modest *R²* values are consistent with the polygenic and multifactorial nature of periodontal dysbiosis, in which individual explanatory variables generally account for a small proportion of total variance. These effect sizes are comparable to those reported in bacterial community studies of PD; for example, a previous 16S metagenome study found that disease status explained 9.7% of variance in salivary microbiome profiles [45]. Within individual cohorts, we further quantified the influence of disease status on viral composition and found that PD status significantly influenced viral composition in Saito_2022 (saliva) and in all subgingival plaque datasets except Moghadam_2023 (Figure 2E–F). Viral family composition remained dominated by *Siphoviridae*, *Myoviridae* and *Podoviridae* irrespective of disease status or site (Figure 2C–D).

### Identification of PD-associated viral signatures in saliva and subgingival plaque

To identify robust viral biomarkers associated with PD in saliva and subgingival plaque, we began by identifying vOTUs observed in no fewer than three samples in each study. Subsequently, we conducted the Wilcoxon rank-sum test for each study to compare the relative abundance of vOTUs between PD patients and healthy controls. In both saliva and subgingival plaque, we observed a subset of vOTUs with markedly low *P*-values deviating from the null expectation, suggesting genuine disease associations (Figure S2E-F). vOTUs showing consistent directional changes with *P* < 0.05 in at least two cohorts were retained as candidates, yielding 202 salivary and 123 subgingival vOTUs.

### Meta-analysis identifies conserved PD-associated vOTUs

To account for inter-study heterogeneity, we applied random-effects meta-analysis separately to each site. This identified 105 salivary and 66 subgingival vOTUs with significant pooled effect sizes (BH-adjusted *P* < 0.05) (Figure S2A–B). For saliva, Hedges' g ranged from −1.30 to 1.47, with all 95% confidence intervals (CIs) excluding zero; heterogeneity was low to moderate (median I² = 15.3%, range 0–77.4%), indicating consistent abundance shifts across studies. For subgingival plaque, effect sizes ranged from −1.52 to 1.16 (all 95% CIs excluding zero), with similar heterogeneity (median I² = 10.3%, range 0–62.44%) (Table S2).

To address potential confounding arising from the lack of explicit diagnostic criteria in the Saito_2022 cohort, we performed a sensitivity analysis. We re-ran the random effects meta-analysis for the 105 identified salivary vOTUs using only the remaining three cohorts (Belstrøm_2017, Belstrøm_2021 and Moghadam_2023) with available diagnostic criteria. After excluding Saito_2022, 53 of the 105 vOTUs (50.5%) remained statistically significant (BH-adjusted *P* < 0.05). The effect sizes of these retained vOTUs were highly concordant with those from the full analysis, indicating that the core viral signatures are robust to the inclusion or exclusion of this cohort. The reduction in number is expected due to the loss of statistical power with fewer cohorts, but the substantial overlap in signal supports the validity of our primary findings.

Permutation testing, which preserves the null distribution while maintaining the complex correlation structure of the data, confirmed the robustness of these findings. The observed number of significant vOTUs (105 saliva, 66 plaque) far exceeded the maximum obtained in 1,000 permutations (57 and 24, respectively; empirical *p* < 0.001 for both) (Figure S2C-D). These results, together with the exclusion of zero from all 95% CIs and the low-to-moderate heterogeneity across cohorts, provide strong evidence that the identified viral signatures are genuine and not artifacts of overfitting or inadequate sample size.

## Confounding by age and sex and cohort contributions

Within cohorts providing individual-level demographic data, we assessed confounding by comparing unadjusted Wilcoxon *p*-values with those from linear models adjusting for age, sex, or both (Table S4). In saliva (Belstrøm_2021, Moghadam_2023), 83.9 and 36.8% of initially significant vOTUs remained robust after full adjustment, respectively. Age exerted a stronger confounding effect than sex, particularly in Moghadam_2023, where age adjustment alone reduced the number of robust vOTUs from 57 to 22 (38.6%), whereas sex adjustment retained 53 (93.0%). In subgingival plaque (Belstrøm_2021, Izawa_2021, Moghadam_2023), 78.9, 32 and 41.2% remained significant after full adjustment, respectively. Again, age showed more pronounced confounding than sex, yet the majority of associations persisted.

The proportion of meta-analysis-derived vOTUs that were independently significant within each cohort varied considerably. In saliva, Saito_2022 and Moghadam_2023 contributed the most (90.5 and 54.3% of vOTUs significant), whereas Belstrøm_2021 contributed least (29.5%) (Figure S2A). In subgingival plaque, Belstrøm_2021 and Wang_2015 showed the highest proportions (86.4 and 72.7%), while Izawa_2021 and Moghadam_2023 contributed modestly (37.9 and 25.8%) (Figure S2B).

### Taxonomic distribution and predicted hosts of PD-associated vOTUs

**Figure 3.**
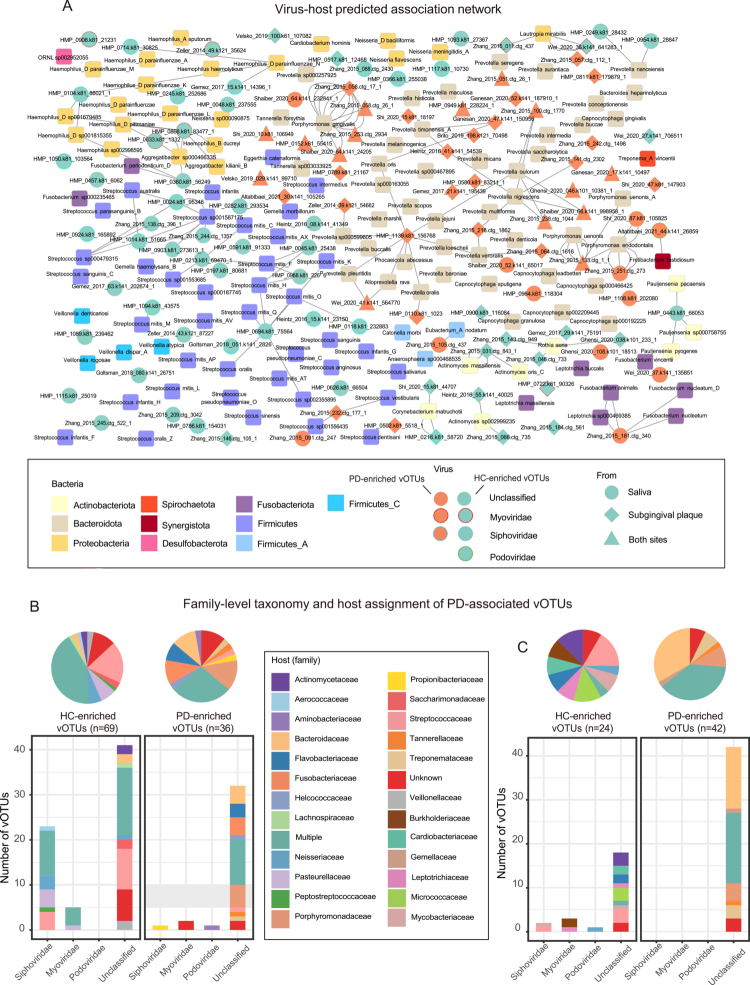
The PD-associated viral signatures. (A) The network was constructed based on virus-host pairs identified through CRISPR-spacer matches and prophage blasts. Nodes represent individual vOTUs (virus nodes) or bacterial taxa at the species level (host nodes); edges indicate predicted infection relationships. Bacterial nodes are colour-coded by phylum, as indicated in the legend. Virus nodes are colour-coded by their abundance pattern in PD: orange for vOTUs enriched in PD, green for vOTUs depleted in PD. Node shapes denote the site of each vOTU: circles represent vOTUs from saliva, diamonds represent vOTUs from subgingival plaque and triangles represent vOTUs shared between both sites. (B–C) The family-level taxonomy and host assignment of PD-associated vOTUs in saliva (B) and subgingival plaque (C). The vOTUs are grouped at the family level, and their hosts are shown at the family level. The numbers of vOTUs that had more than one predicted host are coloured in green (multiple families).

In saliva, 36 of 105 vOTUs were enriched in PD (1 *Podoviridae*, 1 S*iphoviridae*, 2 *Myoviridae* and 32 unclassified), while 69 were enriched in healthy controls (23 S*iphoviridae*, 5 *Myoviridae* and 41 unclassified) (Figure 3B; Table S2). In subgingival plaque, 42 of 66 vOTUs were PD-enriched (all unclassified), and 24 were healthy-enriched (1 *Podoviridae*, 2 *Siphoviridae*, 3 *Myoviridae* and 18 unclassified) (Figure 3C; Table S2). Fifteen vOTUs were shared between the two sites, all PD-enriched.

To investigate the potential ecological roles of PD-associated vOTUs, we performed *in silico* host assignment using CRISPR-spacer matches and prophage homology. Overall, 89.5% of salivary and 92.4% of subgingival PD-associated vOTUs were assigned to one or more putative prokaryotic hosts, yielding 847 virus-host predictions for the 156 vOTUs (18.2% CRISPR-based, 81.7% prophage-based). CRISPR spacer matches provide direct evidence of past viral encounters and thus represent higher confidence host assignments, whereas prophage-based predictions offer broader coverage but rely on sequence similarity that may not indicate active infection cycles.

In saliva, the predicted bacterial hosts of vOTUs differed substantially between the PD-enriched and PD-reduced groups at both family and genus levels. At the family level, 27.8% of PD-enriched vOTUs were predicted to associate with multiple hosts, predominantly targeting *Bacteroidaceae* (9 vOTUs), *Porphyromonadaceae* (8 vOTUs), *Fusobacteriaceae* (5 vOTUs) and *Tannerellaceae* (2 vOTUs). In contrast, 42% of PD-reduced vOTUs showed multi host predictions, primarily involving *Streptococcaceae* (29 vOTUs), *Pasteurellaceae* (13 vOTUs) and *Fusobacteriaceae* (11 vOTUs), and *Aerococcaceae* (7 vOTUs) (Figure 3B; Table S2). At the genus level, PD-enriched vOTUs were predicted to target mainly *Prevotella* (9 vOTUs), *Porphyromonas* (8 vOTUs), *Fusobacterium* (5 vOTUs) and *Tannerella* (2 vOTUs), whereas PD-reduced vOTUs were predicted to predominantly target *Streptococcus* (29 vOTUs), *Fusobacterium* (11 vOTUs) and *Granulicatella* (7 vOTUs) (Table S2).

In subgingival plaque, distinct host prediction patterns were similarly observed. At the family level, 38% of PD‑enriched vOTUs were predicted to associate with multiple hosts, predominantly targeting *Bacteroidaceae* (27 vOTUs), *Porphyromonadaceae* (6 vOTUs), *Treponemataceae* (4 vOTUs) and *Tannerellaceae* (2 vOTUs). Conversely, only 4.2% of PD‑reduced vOTUs exhibited multi‑host predictions, primarily involving *Streptococcaceae* (4 vOTUs), *Flavobacteriaceae* (3 vOTUs) and *Micrococcaceae* (3 vOTUs) (Figure 3B; Table S2). At the genus level, PD‑enriched vOTUs were predicted to target primarily *Prevotella* (27 vOTUs), *Porphyromonas* (6 vOTUs) and *Tannerella* (2 vOTUs), while PD‑reduced vOTUs were predicted to predominantly target *Streptococcus* (4 vOTUs), *Capnocytophaga* (3 vOTUs) and *Rothia* (3 vOTUs) (Table S2).

To further illustrate these relationships, we constructed a virus-host prediction network based on direct genomic evidence, with bacterial hosts resolved at the species level. The network comprised 431 edges connecting 113 out of 156 vOTUs and 133 bacterial taxa (Figure 3A). Examination of the predicted hosts for PD-enriched vOTUs revealed frequent targeting of well-established periodontal pathogens, including *Porphyromonas gingivalis, Tannerella forsythia*, *Prevotella intermedia* and *Fusobacterium nucleatum* [46-48]. Previous studies have shown that the abundance of these four bacterial species in subgingival plaque increases with the progression of periodontal inflammation [49].

### Genome characteristics of PD-associated vOTUs

**Figure 4.**
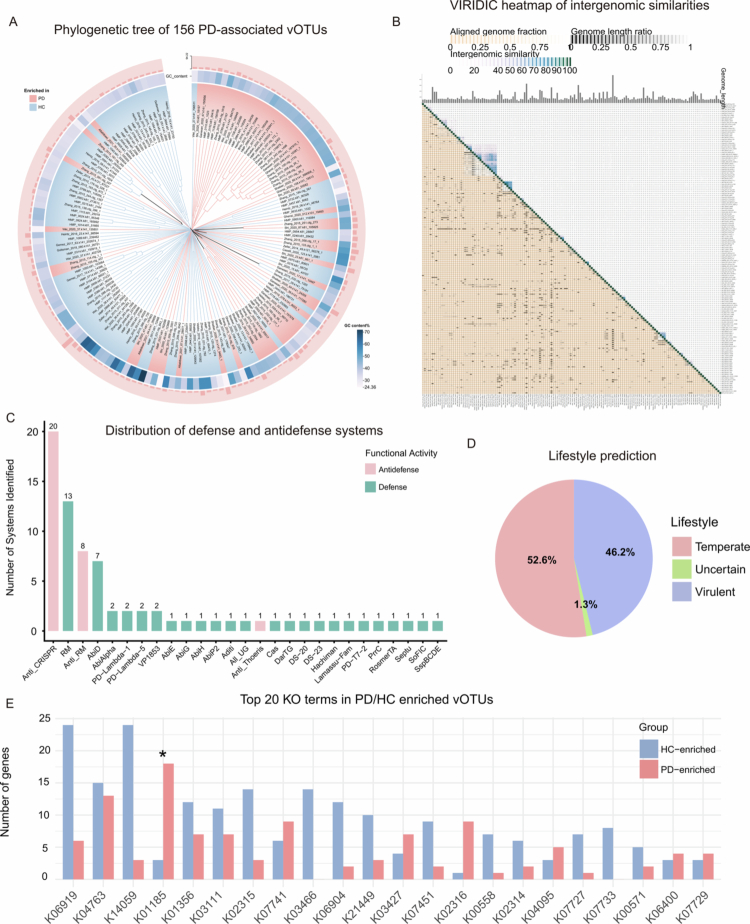
The genome analysis and function annotation of PD-associated vOTUs. (A) Maximum-likelihood phylogenetic tree of 156 vOTUs. The colored outer rings represent differential abundance direction in PD, with red indicating enrichment in PD and blue indicating enrichment in HC. The gradients of GC content are shown in blue, where darker blue indicates higher GC content. The pale red outer bars indicate the length of vOTUs, with the length presented in units of kilobases (kb). (B) VIRIDIC-generated heatmap of intergenomic similarities. Right matrix: Similarity values (darker hues = higher similarity; values rounded to 1 decimal). Left/top annotations: Alignment quality (orange‒white gradient = alignment fraction; black‒white gradient = genome length differences). (C) Different numbers of defense and antidefense systems were identified in 156 vOTUs using DefenseFinder. (D) Lifestyle prediction results (temperate or virulent) of 156 vOTUs. (E) KEGG enrichment analysis of PD-associated vOTUs. The bar plot displays the top 20 most abundant KO terms among PD-reduced (blue) or PD-enriched (red) vOTUs. Counts indicate the number of genes per KO term. Asterisks (*) denote significant enrichment (Fisher's exact test, BH-adjusted *P* < 0.05).

The 156 PD-associated vOTUs exhibited substantial genomic diversity, with GC content ranging from 24.4 to 71.9% and genome lengths spanning 6.26–94.22 kb. Based on CheckV completeness estimates, 28 vOTUs (17.9%) were classified as high-quality, 17 (10.9%) as medium-quality, 110 (70.5%) as low-quality and one (0.6%) as undetermined-quality (Table S2). Phylogenetic analysis of all 156 PD-associated vOTUs revealed substantial genetic diversity (Figure 4A), and pairwise intergenomic similarity analysis using VIRIDIC demonstrated that most vOTU pairs shared low sequence identity, indicating considerable sequence divergence. A small subset of vOTUs formed clusters with high similarity, suggesting the presence of conserved viral lineages within the broader genetic diversity (Figure 4B).

### Function annotation of PD-associated vOTUs

Lifestyle prediction classified the 156 PD-associated vOTUs as 52.6% temperate, 46.2% virulent and 1.3% uncertain (Figure 4D; Table S2). The prevalence of temperate phages suggests a propensity for lysogeny within the periodontal niche, potentially facilitating horizontal gene transfer and long-term genetic integration into bacterial host genomes.

KEGG enrichment analysis revealed significant enrichment of specific functionally annotated genes in PD-enriched vOTUs, most notably lysozyme (K01185) (BH-adjusted *P* < 0.05). Enrichment trend was also observed for DNA primase (K02316). In contrast, the putative DNA primase/helicase (K06919), which was the most abundant KO term overall, showed a trend toward enrichment in HC-enriched vOTUs (Figure 4E; Table S3). Lysozyme facilitates progeny release via bacterial cell wall degradation, and its enrichment in PD suggests enhanced lytic activity within the diseased periodontium. The parallel trend for DNA primase supports increased viral replication potential. Conversely, the skew of the broadly distributed primase/helicase toward health implies a partitioning of replication-associated functions between disease- and health-linked viral populations. This functional divergence may reflect altered viral life cycle strategies under inflammation, potentially influencing virus-host turnover and contributing to periodontal dysbiosis.

Profiling of defence and anti-defence systems using DefenseFinder identified both bacterial defence modules and viral counter-defence factors within PD-associated vOTUs. The most abundant anti-defence genes were Anti-CRISPR (*n* = 20), followed by Anti-RM (*n* = 8) (Figure 4C; Table S3). The presence of both defence and counter-defence genes points to an ongoing molecular arms race between phages and their bacterial hosts, representing a potential adaptive strategy for viral persistence within the periodontal niche.

### Predictive modelling of PD using viral signatures

**Figure 5.**
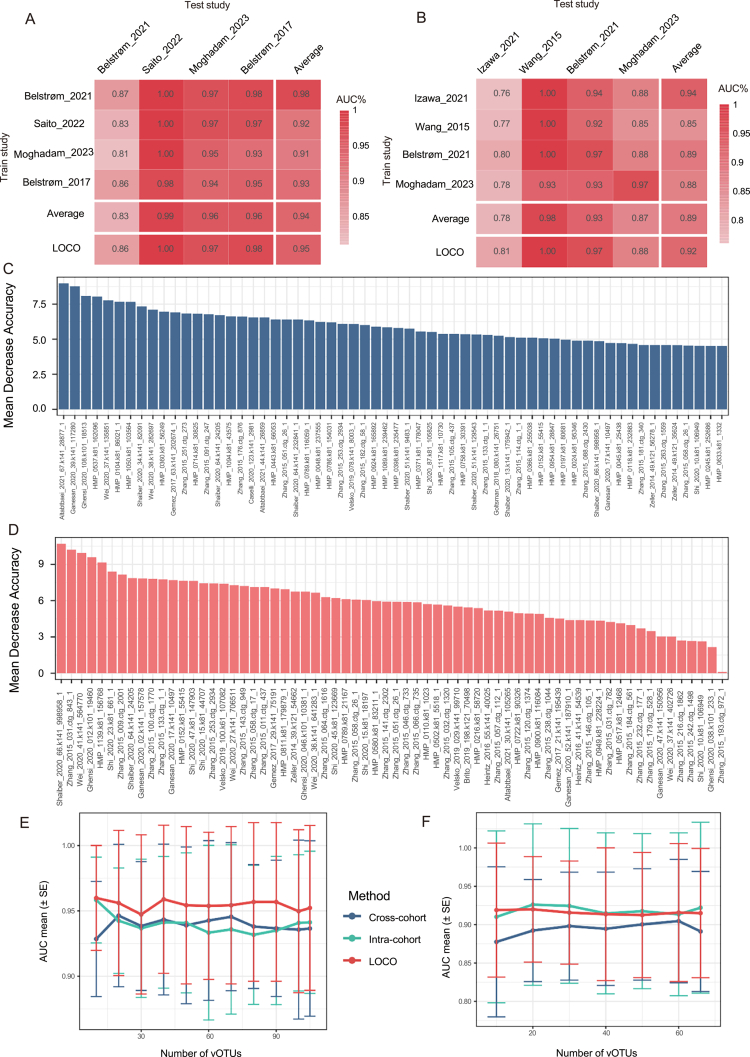
The random forest prediction model of PD status based on relative abundances of 105 PD-associated vOTUs in saliva and 66 in subgingival plaque. (A to B) Performance assessment as AUC scores of intra-dataset, cross-dataset and LOCO predictions using random forest models in predicting PD status in saliva (A) and subgingival plaque (B). The model of intra-dataset prediction (diagonal) was validated using five repeats of fivefold cross-validations. The model of cross-dataset prediction (non-diagonal) was built on the dataset corresponding to each row and validated on the dataset corresponding to each column. The LOCO row refers to Leave-One-Cohort-Out analysis in which models were built on three datasets combined and validated on the remaining one corresponding to each column. Average refers to the mean of non-diagonal (cross-cohort). (C–D) Global feature importance calculated by the ‘mean decrease accuracy’ method of top 60 PD-associated vOTUs in saliva (C) and all 66 in subgingival plaque (D). (E–F) Average AUC values for different numbers of PD-associated vOTUs in saliva (E) and subgingival plaque (F) using random forest models.

To evaluate the diagnostic potential of oral viral signatures, we constructed random forest models using the 105 salivary and 66 subgingival PD-associated vOTUs. Within-dataset cross-validation yielded mean AUCs of 0.94 for saliva (range 0.87–1.00) and 0.92 for subgingival plaque (range 0.76–1.00) (Figure 5A,B, diagonal). Cross-dataset validation, wherein models were trained on one cohort and tested on the remaining three, produced mean AUCs of 0.94 for saliva (range 0.81–1.00) and 0.89 for subgingival plaque (range 0.77–1.00) (Figure 5A,B, off-diagonal). To further assess generalisability, we performed LOCO analysis, iteratively training on three datasets and validating on the withheld cohort. LOCO validation achieved mean AUCs of 0.95 for saliva (range 0.86–1.00) and 0.92 for subgingival plaque (range 0.81–1.00) (Figure 5A,B).

In addition to AUC, we evaluated a comprehensive set of performance metrics for each LOCO validation to provide a more complete assessment of classifier performance, particularly given the imbalanced class distributions in some cohorts. These metrics, including accuracy, sensitivity, specificity, positive predictive value (PPV), negative predictive value (NPV) and F1 score, are reported in Table S4, along with the corresponding confusion matrices. Performance varied across cohorts, reflecting the heterogeneity inherent in multi-cohort studies. For saliva, LOCO validation yielded sensitivity ranging from 0.64 to 1.00 and specificity from 0.73 to 1.00, with F1 scores from 0.74 to 1.00. For subgingival plaque, sensitivity ranged from 0.70 to 1.00 and specificity from 0.83 to 1.00, with F1 scores from 0.78 to 0.91 (Table S4). The lowest saliva LOCO sensitivity (0.64) was recorded for the Belstrøm_2021 cohort. Notably, this cohort exhibited the fewest within‑cohort significant vOTUs among the four saliva datasets (31 of 105, compared with 47, 57 and 95 in Belstrøm_2017, Moghadam_2023 and Saito_2022, respectively), indicating a relatively attenuated viral signature. Importantly, the model maintained high specificity (0.91) and an AUC of 0.86, underscoring that the viral signature retains meaningful discriminatory capacity even under these conditions. Despite this variability, the overall performance metrics across LOCO iterations corroborated the AUC findings, confirming that the viral signatures maintain robust discriminatory power when tested on completely unseen cohorts.

Feature importance was assessed using mean decrease in accuracy (MDA) (Figure 5C,D). To identify minimal robust signature sets, we evaluated model performance using progressively smaller feature subsets. In saliva, the top 10 vOTUs by MDA achieved a mean LOCO AUC of 0.96 (Figure 5E, Figure S3F). In subgingival plaque, the top 20 vOTUs yielded a mean LOCO AUC of 0.92 (Figure 5F, Figure S4E). These results indicate that a reduced set of viral biomarkers retains high discriminatory power, supporting their potential utility for diagnostic applications.

## Discussion

Although PD is well established as a chronic infectious disease triggered by dysbiotic bacterial biofilms, the majority of studies have focused on bacterial communities [50,51], with limited exploration of the oral virome [9,10]. In our study, we characterised the oral viral communities by generating a profile of 37,692 vOTUs from 234 samples across six published cohorts, of which 58.9% were assigned to known viral families, predominantly comprising *Siphoviridae*, *Myoviridae* and *Podoviridae*. It should be noted that these taxonomic assignments follow the traditional ICTV framework, which was superseded in 2022 by a phylogeny-based taxonomy within the class *Caudoviricetes*. While this revision does not alter the viral sequences themselves or their observed associations with PD, it has implications for cross-study comparisons. Future studies should adopt the updated ICTV taxonomy to ensure consistent and meaningful comparisons across oral virome datasets. We further observed the viral alpha diversity metrics were highly concordant with those of the corresponding bacterial communities (Figure S1E–F). This concordance likely reflects the predominantly bacterial host range of the detected viruses and aligns with the notion that periodontal inflammation fosters increased bacterial load and diversity, consequently shaping the associated viral community.

Through meta-analysis of four independent cohorts per site, we identified 105 salivary and 66 subgingival vOTUs robustly associated with PD. Lifestyle prediction classified 52.6% of these vOTUs as temperate; however, the accuracy of BACPHLIP may be compromised when applied to fragmented metagenomic contigs, as it is primarily trained on complete phage genomes. Notably, the proportion of temperate predictions was markedly higher among high-quality vOTUs (82.1%) than among Low-quality ones (45.4%), suggesting that incomplete sequences may bias predictions toward virulent classifications. This discrepancy likely arises because fragmented genomes often lack key lysogeny-associated genes (e.g. integrases) or contain truncated marker genes, leading to misclassification. When restricting the analysis to Medium and high-quality vOTUs only, the overall proportion of temperate phages was 71.1%, providing a more reliable estimate of the true lifestyle distribution within this dataset. The predominance of temperate phages within the reliable subset robustly supports our conclusion that temperate lifestyles are prevalent among PD-associated viruses.

Virus-host prediction revealed a striking pattern: vOTUs enriched in PD were predicted to target well-established periodontal pathogens, including *Porphyromonas gingivalis*, *Tannerella forsythia*, *Prevotella intermedia* and *Fusobacterium nucleatum* [46-48] (Figure 3A). This association can be interpreted through at least two non-mutually exclusive ecological frameworks. First, temperate phages integrated as prophages may be induced under inflammatory conditions, simultaneously increasing viral particle abundance and potentially enhancing bacterial virulence through lysogenic conversion. Second, virulent phages could exploit the elevated density of susceptible hosts in dysbiotic biofilms, leading to concomitant increases in both populations without necessarily implying direct predation. Critically, this virus-host pattern is not unique to PD. A recent study of childhood dental caries revealed phage-bacterial associations with health and disease: several vOTUs predicted to infect *Haemophilus* and *Neisseria* were strongly correlated with health, whereas five vOTUs predicted to infect *Saccharibacteria*, *Prevotella* and *Veillonella* were correlated with caries [52]. These findings suggest conserved ecological principles underlying phage-driven dysbiosis in oral diseases. Conversely, we observed decreased levels of phages predicted to target *Streptococcus* in PD patients, suggesting that depletion of phages targeting health-associated taxa may represent another facet of viral dysbiosis.

Our random forest models demonstrated robust discriminatory performance across multiple validation strategies, with LOCO validation achieving mean AUCs of 0.95 for saliva and 0.92 for subgingival plaque. These results are comparable to recently published models based on PD bacterial biomarkers [53]. In subgingival plaque, *Shaiber_2020_66.k141_998958_1* emerged as the top contributor (Figure 5D). In saliva, *Altabtbaei_2021_67.k141_28877_1,* a PD-enriched vOTU, showed the highest feature importance and *Ganesan_2020_39.k141_117280* predicted to target multiple hosts showed the second highest feature importance (Figure 5C). Such multi-host predictions may reflect genuine biological promiscuity or technical artifacts (e.g. database redundancy), and require experimental validation.

Notably, certain cohorts, such as Saito_2022 (saliva) and Wang_2015 (subgingival plaque), achieved perfect or near-perfect AUCs (1.00) when serving as validation sets. While highlighting strong disease-associated viral signals, such values warrant caution. They may reflect both true biological alignment with the meta-analysis-derived biomarkers and the optimistic bias introduced by small, homogeneous cohorts. Critically, the LOCO framework precludes data leakage, confirming that these AUCs reflect genuine generalisability rather than overfitting. Cohort-specific contributions to the differentially abundant vOTU set mirror variation in demographics, disease severity and technical protocols. Nonetheless, the integrated random forest classifier maintained robust cross-cohort performance, indicating that the composite viral signature captures a stable, PD-associated biological pattern despite substantial clinical heterogeneity across studies.

Currently, initial non-surgical periodontal therapy primarily involves home care review, scaling and root planning and for residual sites with active PD during periodontal re-evaluation, contemporary regenerative or traditional surgical therapy may be utilised [54]. The use of bacteriophages as antibacterial agents represents a promising frontier in the future treatment of PD due to their specificity, ability to disrupt biofilms and rapid propagation capabilities [55]. Our finding that PD-enriched vOTUs frequently target keystone pathogens provides biological rationale for exploring phage-based interventions. The association between PD and increased abundance of phages targeting Porphyromonas gingivalis and other pathogens may reflect prophage induction under inflammatory conditions or enhanced lytic replication within dense pathogen populations. Understanding whether these phages exert protective or pathogenic effects will require experimental dissection of the interplay between phage predation, bacterial counter-adaptation and host immunity.

Several limitations of this study warrant consideration. First, a key constraint arises from the meta-analytic design: the integrated cohorts were not originally designed for pooled analysis, resulting in heterogeneity in clinical definitions of PD and incomplete recording of key phenotypic data (e.g. smoking status, diabetes, detailed medication history). Among these, the absence of smoking status is particularly notable, as smoking is a well-established risk factor for PD and is known to independently alter the composition of the oral microbiome; unmeasured confounding by smoking may therefore influence the strength or specificity of the reported viral associations. Critically, the absence of a uniformly applied modern diagnostic standard (e.g. AAP/EFP 2018 [56] fundamentally hampers the interpretation of diagnostic performance metrics, as noted in recent methodological critiques [57]. This heterogeneity is particularly relevant for the Saito_2022 cohort, for which explicit diagnostic criteria were not reported; although its case‒control design support inclusion, the lack of standardised definitions introduces uncertainty regarding generalisability. The combination of limited per-cohort sample sizes and a high-dimensional feature space also raises concerns about potential model overfitting. Although LOCO validation was employed to test generalisability, we cannot fully exclude the influence of unmeasured confounders or technical biases that may correlate with cohort identity. Therefore, the diagnostic performance observed here should be viewed as a robust, yet provisional, estimate that requires validation in future prospective studies. Second, our reliance on read-mapping to the OVD rather than *de novo* assembly enabled standardised characterisation but inherently precluded discovery of novel viral genomes absent from existing reference databases. Consequently, our catalogue of PD-associated phages is likely incomplete, potentially overlooking previously uncharacterised viral lineages that may play important roles in periodontal dysbiosis. Third, our cross-sectional metagenomic data reflect viral genome abundance but cannot demonstrate activity, lysogeny status, or causal directionality in virus-host interactions. The multi-host predictions for certain vOTUs may represent genuine biological promiscuity or technical artifacts, and our reliance on direct genomic evidence, while ensuring high confidence, may overlook interactions lacking such evidence. Future studies using integrative tools like iPHoP [58] could help expand the predicted interaction network, but experimental validation remains essential to confirm biological specificity. Fourth, our bioinformatic pipeline was optimised for bacteriophage detection and does not comprehensively capture eukaryotic viruses, whose potential contributions to the periodontal ecosystem remain unassessed.

Future work should prioritise prospective, longitudinal cohorts diagnosed with standardised clinical criteria (e.g. AAP/EFP 2018), enabling adjustment for key confounders and improved biomarker validation. Beyond descriptive associations, incorporating metatranscriptomics could help differentiate active viral replication from lysogeny, while experimental models are essential to move from correlation to causation and elucidate the specific roles of phages in periodontal pathogenesis. Ultimately, studies employing harmonised diagnostic criteria in larger multicenter cohorts are necessary to validate the specificity and sensitivity of the proposed viral signatures across different populations and diagnostic frameworks.

Despite these limitations, our study establishes that reproducible viral signatures are associated with PD across heterogeneous cohorts. The enrichment of phages targeting keystone pathogens, together with their genomic capacity for integration and host modulation, positions the oral virome as a dynamic and underexplored component of the periodontal ecosystem. These findings underscore the oral virome as a source of novel biomarkers and provide a foundation for future mechanistic and therapeutic investigations.

## Conclusions

We conducted a comprehensive assessment of changes in oral viral composition in patients with PD and described the potential interactions between viral biomarkers and bacteria. Although the exact mechanism by which these viruses contribute to the onset of PD remain unclear, our work presents several plausible explanations. Furthermore, phages hold significant potential for the treatment of PD [55]. Our analysis strongly indicates the need for further research to elucidate the precise role of overlooked oral viruses in PD development and to explore new phage therapies as potential treatments for PD.

## Supplementary Material

Supplementary Material Supplement material.docx

## Data Availability

Raw metagenomic data are available in the NCBI SRA under the accession numbers listed in Table 1. The oral virus database (OVD) is available at https://github.com/RChGO/OVD. All vOTU abundance matrices, PD-associated viral sequences and random forest codes in this study are deposited at https://github.com/CCCts123/PD_virome. The authors declare that all other data supporting the findings of this study are available in the paper and supplementary materials, or can be obtained from the corresponding authors upon request.
