# Supplementary material for: Metagenome-based virome analysis identifies the oral viral signatures for periodontitis
Source: J Oral Microbiol. 2026 Apr 25;18(1):2662091. doi: 10.1080/20002297.2026.2662091 (PMC13112875; doi:10.1080/20002297.2026.2662091)
Supplement: Supplementary Material — Supplement material.docx [file ZJOM_A_2662091_SM2534.docx]

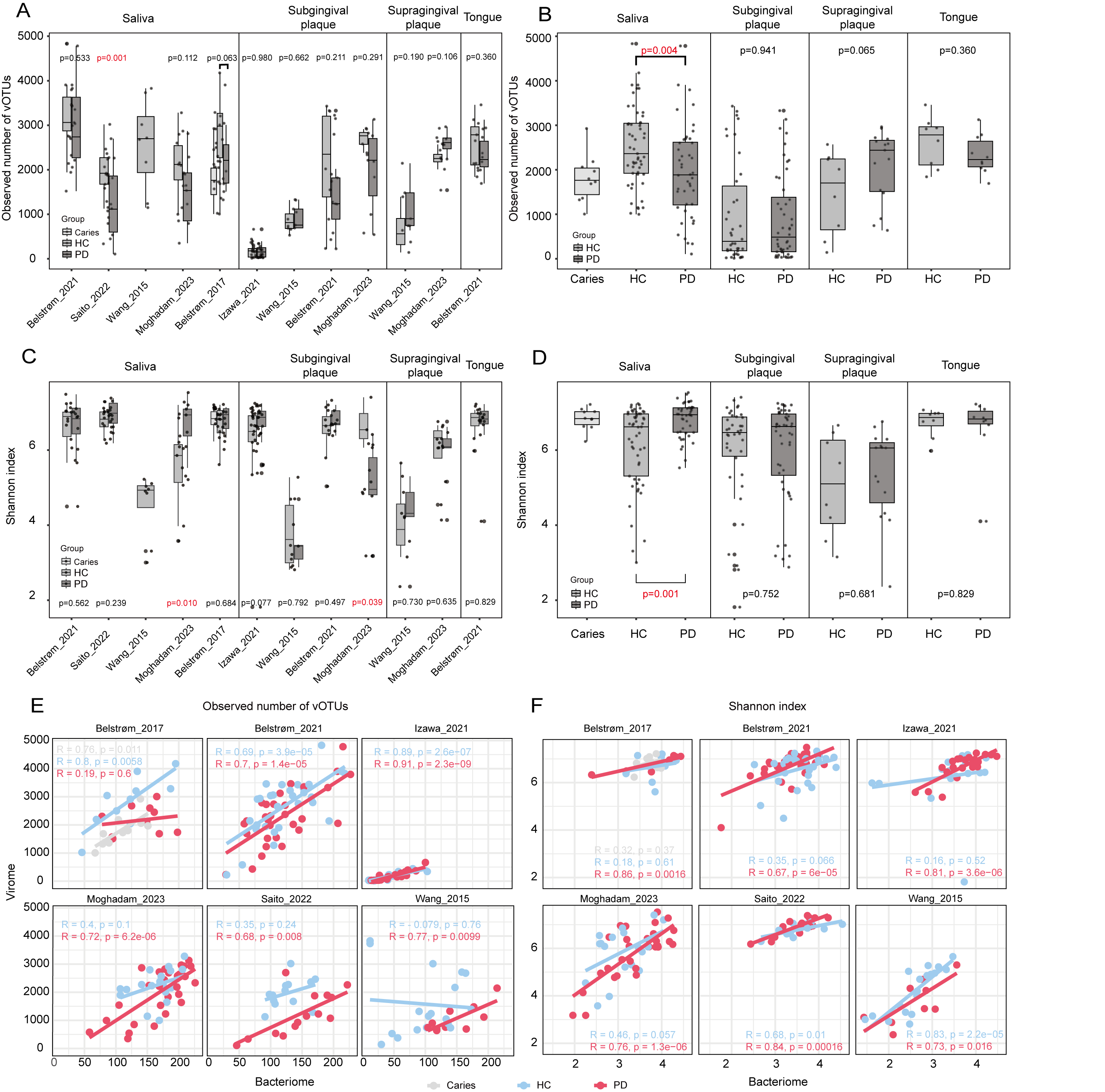


**Fig. S1: Species richness and uniformity of the oral microbiome in the PD dataset. (A)** Comparison of the observed number of vOTUs among three groups in each dataset. Significance test was performed by two-tailed Wilcoxon rank sum test. HC, healthy controls; PD, periodontitis patients; Caries, caries patients. **(B)** Comparison of the observed number of vOTUs among three groups when combining studies from all datasets. **(C)** Comparison of Shannon diversity index between two groups in each dataset. **(D)** Comparison of Shannon diversity index between two groups when combining studies from all datasets. **(E)** Scatter plots of the observed number of vOTUs of virome and bacteriome in each dataset. The correlation of alpha diversity between virome and bacteriome was shown by linear regression line. The correlation test was performed by Pearson rank correlation method. **(F)** Scatter plot of Shannon diversity index of virome and bacteriome in each dataset.


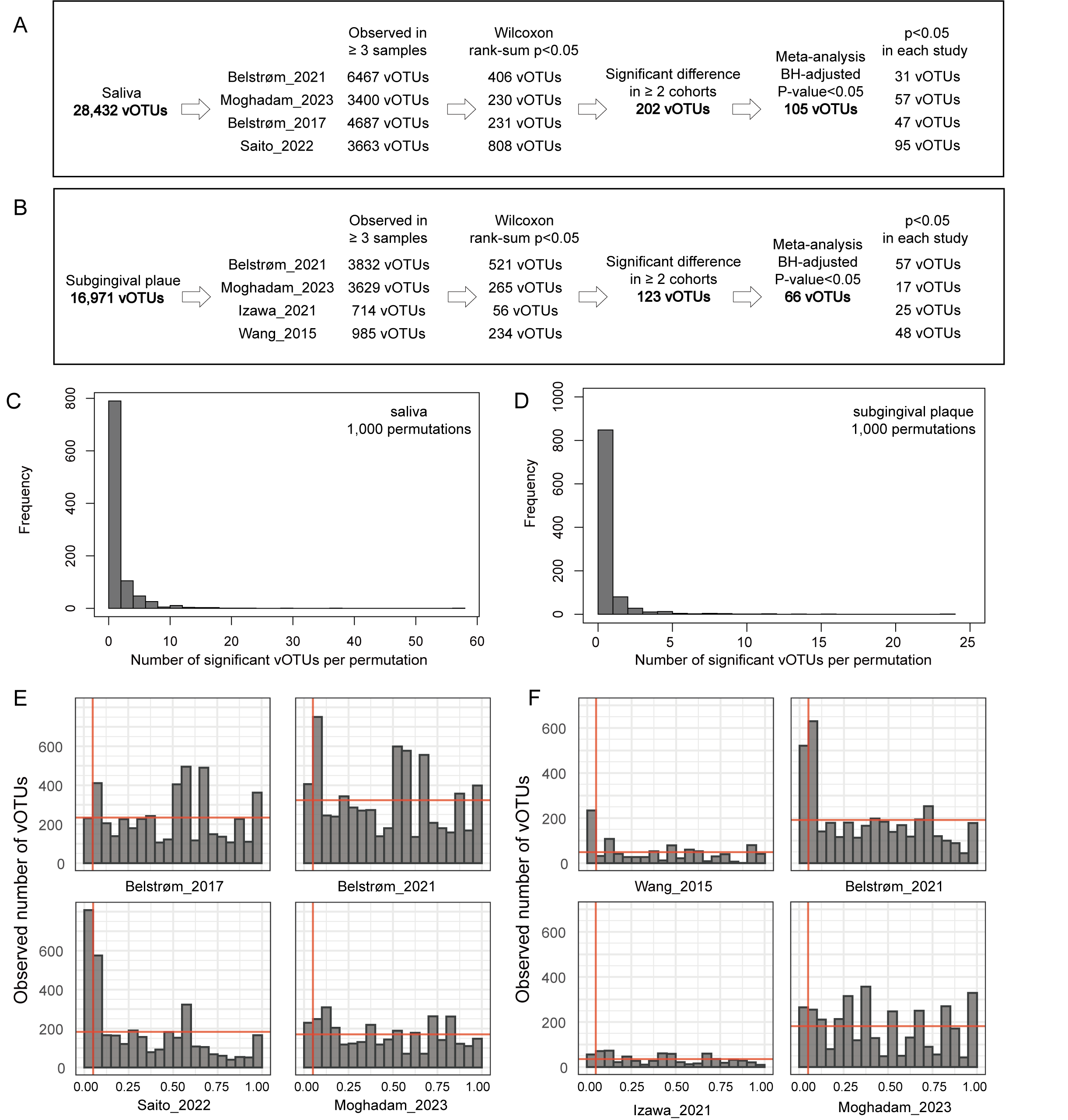


**Fig. S2: PD-associated viral biomarkers identification across four studies in saliva and four studies in subgingival plaque. (A-B)** The workflow for identifying PD-associated viral biomarkers in saliva (A) and subgingival plaque (B). **(C-D)** Permutation test results for (C) saliva and subgingival plaque (D). Histograms show the null distributions of the number of significant vOTUs obtained from 1,000 permutations, generated by randomly shuffling disease/healthy labels within each cohort and applying the full two-stage selection procedure. **(E-F)** The distribution of p-values from the Wilcoxon rank-sum test for the relative abundance of vOTUs in each case-control study (healthy controls versus periodontitis) for saliva (E) and subgingival plaque (F). The red X-axis denotes a p-value of 0.05, while the red Y-axis represents the expected distribution of p-values under the null hypothesis.


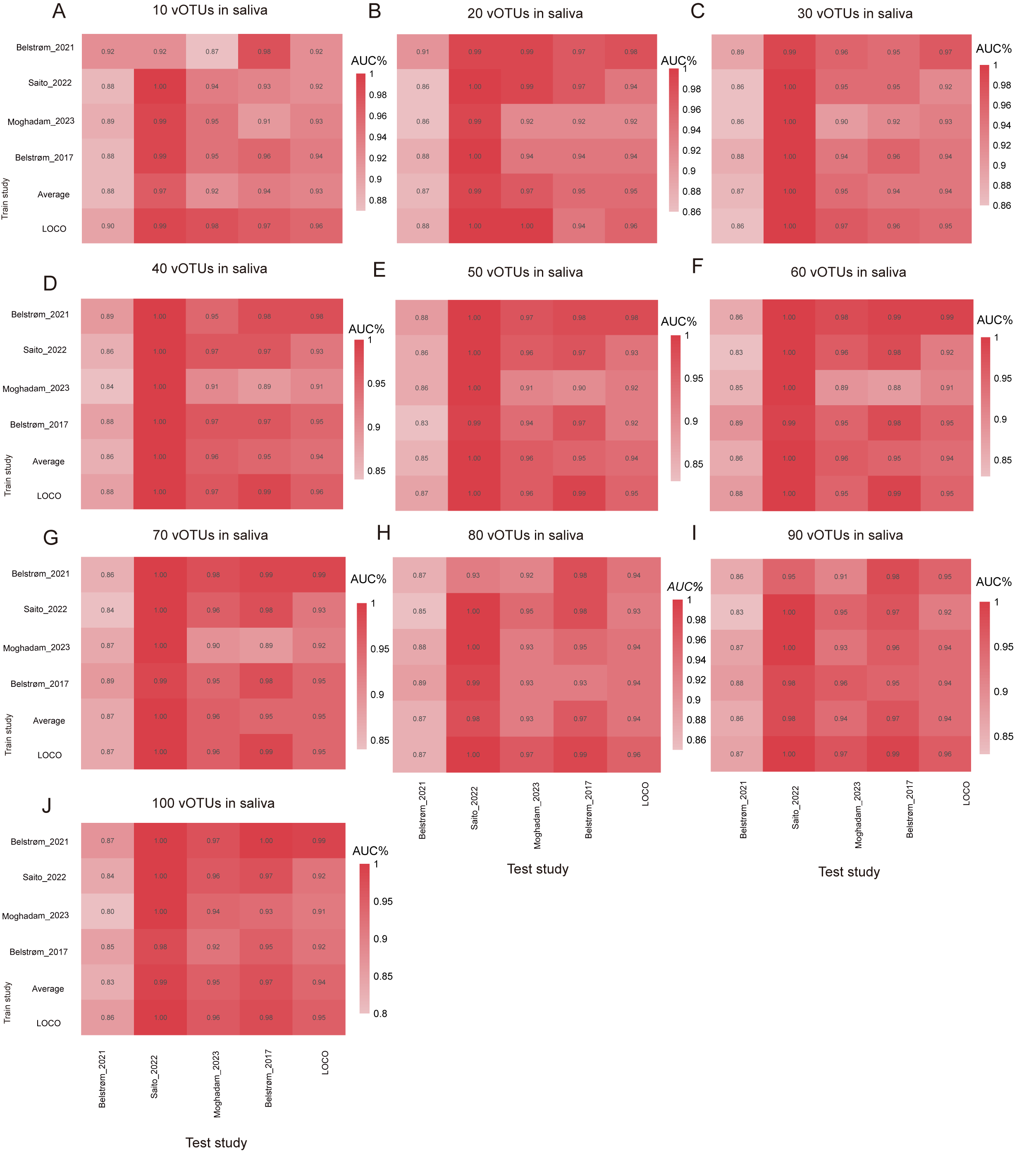


**Fig. S3: Performance assessment as AUC scores for different numbers of PD-associated vOTUs in saliva using random forest models in predicting PD status.** The model of intra-dataset prediction (diagonal) was validated using five repeats of fivefold cross-validations. The model of cross-dataset prediction (non-diagonal) was built on the dataset corresponding to each row and validated on the dataset corresponding to each column. The LOCO row refers to leave-one-cohort-out (LOCO) analysis in which models were built on three datasets combined and validated on the remaining one corresponding to each column. Average refers to the mean of non-diagonal (cross-cohort)


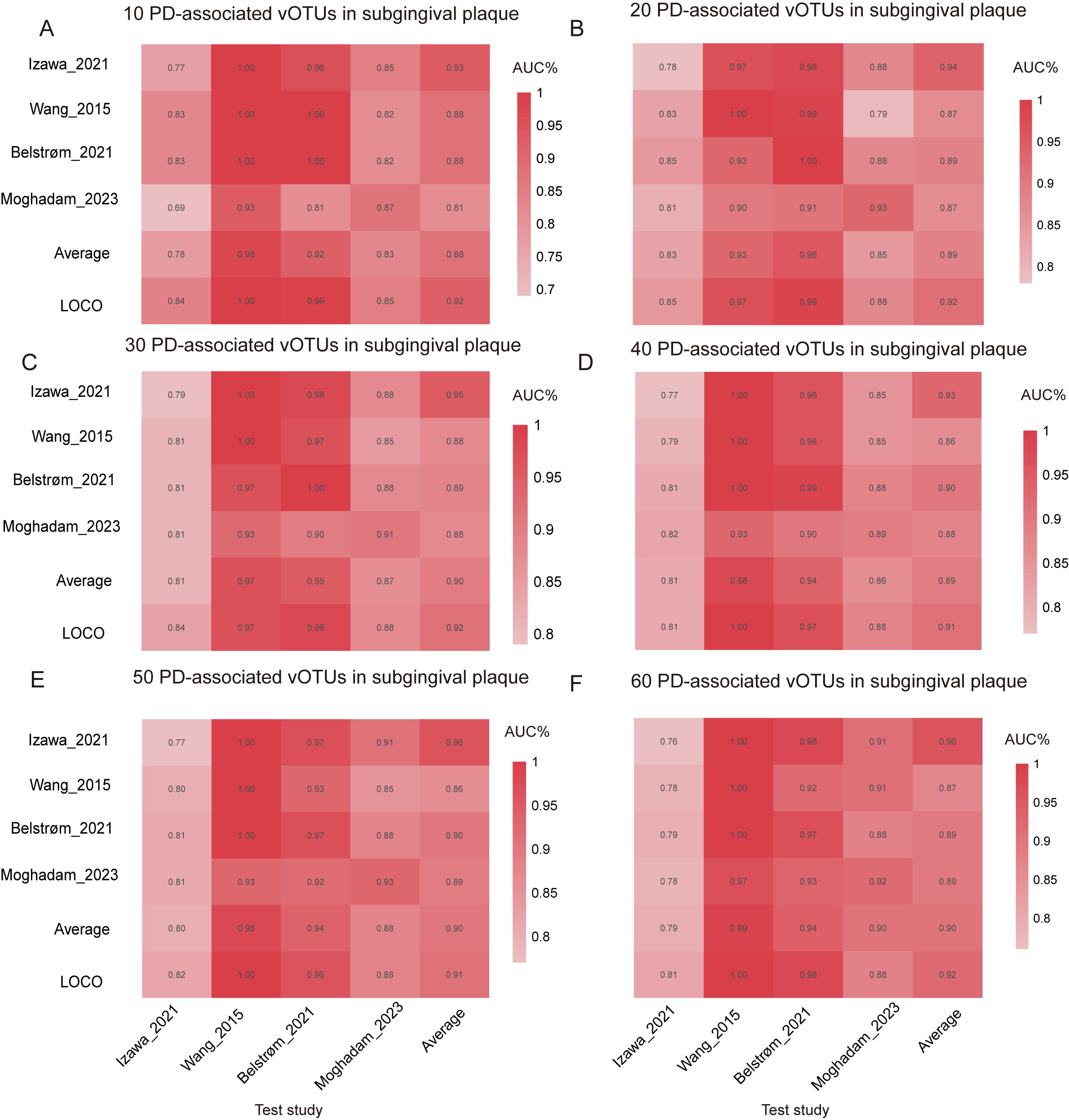


**Fig. S4: Performance assessment as AUC scores for different numbers of PD-associated vOTUs in subgingival plaque using random forest models in predicting PD status.** The model of intra-dataset prediction (diagonal) was validated using five repeats of fivefold cross-validations. The model of cross-dataset prediction (non-diagonal) was built on the dataset corresponding to each row and validated on the dataset corresponding to each column. The LOCO row refers to leave-one-cohort-out (LOCO) analysis in which models were built on three datasets combined and validated on the remaining one corresponding to each column. Average refers to the mean of non-diagonal (cross-cohort)
